# Findable Accessible Interoperable Re-usable (FAIR) diffraction data are coming to protein crystallography

**DOI:** 10.1107/S2052252519005918

**Published:** 2019-04-30

**Authors:** John R. Helliwell, Wladek Minor, Manfred S. Weiss, Elspeth F. Garman, Randy J. Read, Janet Newman, Mark J. van Raaij, Janos Hajdu, Edward N. Baker

**Affiliations:** a School of Chemistry, The University of Manchester, Brunswick Street, Manchester M13 9PL, United Kingdom; b Department of Molecular Physiology and Biological Physics, University of Virginia, 1340 Jefferson Park Avenue Pinn Hall, Charlottesville, VA 22908-0736, USA; c Macromolecular Crystallography (HZB-MX), Helmholtz-Zentrum Berlin, Albert-Einstein-Str. 15, D-12489 Berlin, Germany; d Department of Biochemistry, University of Oxford, South Parks Road, Oxford OX1 3QU, United Kingdom; e Cambridge Institute for Medical Research, Department of Haematology, University of Cambridge, The Keith Peters Building, Hills Road, Cambridge CB2 0XY, United Kingdom; f Collaborative Crystallisation Centre (C3), CSIRO, 343 Royal Parade, Parkville, VIC 3052, Australia; g CSIC, Centro Nacional de Biotecnologia, c/Darwin 3, Madrid, 28049, Spain; h Laboratory of Molecular Biophysics, Department of Cell and Molecular Biology, Uppsala University, Husargatan 3, Box 596, Uppsala, 75124, Sweden; i The European Extreme Light Infrastructure, Institute of Physics, AS CR, Na Slovance 2, Prague 18221 8, Czech Republic; j School of Biological Sciences, University of Auckland, School of Biological Sciences, Private Bag 92-019, Auckland, New Zealand

**Keywords:** FAIR, diffraction data, IUCr policy

## Abstract

The policy of IUCr Journals on diffraction data is defined.

The unprecedented progress of modern science is driven, to a large extent, by the fast propagation of information. Descriptions of experiments and results, and their interpretation, are no longer disseminated solely in peer-reviewed scientific publications, but are frequently distributed through non-reviewed publication platforms as preprints, entries to data repositories, databases *etc*. As a result of ever faster computers and internet connections, many experimental results are now available instantaneously at the click of a mouse, irrespective of the location of the source or consumer.

In many instances, experiments performed and interpreted by one scientific group stimulate the interest of other scientists enough to spur research in further laboratories. Not infrequently, the results of these follow-up experiments are in disagreement with the previously obtained results and/or interpretations (Baker, 2016 ▸), notably in psychology and the clinical sciences. In some cases, the original results cannot even be reproduced well enough to allow follow-up experiments to commence (Prinz *et al.*, 2011 ▸).

Repeating an entire experiment performed by others is usually not feasible because of the significant time, effort and funds it would require (Baker, 2015 ▸). So the question is, what should be done in this new era? How can new technical developments be best exploited for furthering science and the scientific output?

The structural biology community has always been at the forefront of sharing processed, *i.e.* analysed, results. Since its creation in 1971, the Protein Data Bank (PDB; Berman *et al.*, 2000 ▸) has become an indispensable daily resource for hundreds of thousands of scientists. Initially, the PDB curated only the molecular structure coordinate files, but since 2008 the deposition of the processed diffraction data, *i.e.* intensities or structure-factor amplitudes, has been mandatory for each derived coordinate set. At present, all serious scientific journals require the deposition of the coordinates of the structures and the associated diffaction data as well as the submission of a PDB validation report with the manuscript for review. Notable also is a recent initiative by *Science* of the introduction of a Statistical Board of Reviewing Editors (McNutt, 2014*a* ▸,*b* ▸). This is an initiative similar to the practice of some referees insisting on access to the underpinning crystallographic data (Helliwell, 2018 ▸). Certainly, the PDB is an indispensable resource not only for structural biology but for all modern biological, biomedical and biochemical science (Burley *et al.*, 2019 ▸).

However, even with diffraction data being a part of every macromolecular crystallographic deposition in the PDB, and even assuming ‘perfect’ data reduction and processing of the original diffraction images, some experimental information, *e.g.* diffuse scattering, is irrevocably lost. Moreover, our experience shows that quite often, the processing of diffraction data images is far from being perfect: the diffraction data could be processed to higher resolution as software improves, data are sometimes processed in an incorrect space group, the correction for radiation decay may not be optimal, corrupted images can be used during processing, instrument malfunctions are not identified *etc*. (Zimmerman *et al.*, 2014 ▸). Recovery from such errors is very difficult, sometimes even impossible, and suboptimal, or even incorrect, macromolecular structures are often the result (Weiss *et al.*, 2016 ▸). This can adversely affect subsequent research that uses the structure for data mining, for drug discovery or as a training set for artificial intelligence (AI) programs, for example. An overreliance on the incorrectly processed data in the original publication may mislead or even ruin subsequent research efforts.

Not too long ago, the establishment of a repository of macromolecular crystallography diffraction image data sets was perceived to be a ‘mission impossible’ task, mainly because of the prohibitive cost of storage, but also because of the apparent difficulties in organizing such a repository and validating the metadata describing the experiment (Baker, 2017 ▸). However, in the past few years two initiatives have led to large-scale repositories dedicated to diffraction experiments now being available: the Integrated Resource for Reproducibility in Macromolecular Crystallography (IRRMC, currently with over 3800 experiments and 7000 data sets) (Grabowski *et al.*, 2016 ▸) and the Structural Biology Grid Consortium (SBGrid, currently with 400 diffraction experiments, 500 data sets) (Meyer *et al.*, 2016 ▸). These are complemented by several smaller repositories, measured by the number of data sets available to the public, such as the Australian Store.Synchrotron facility (https://store.synchrotron.org.au/) and the data depository for X-ray lasers (CXIDB, https://www.cxidb.org) which hosts terabyte-range data sets. Universities have also started providing data archives for their researchers, such as the repository at the University of Manchester (http://www.itservices.manchester.ac.uk/ourservices/catalogue/research/servers/archive/). Diffraction image data sets are also deposited in general research data repositories such as Zenodo (https://zenodo.org/). Data sets stored in all these repositories are assigned digital object identifiers or dois, which are widely agreed as a primary requirement.

In 2011, the IUCr established the Diffraction Data Deposition Working Group (DDDWG) in order to ‘address the growing calls within the crystallographic community for the deposition of diffraction data images, with some mechanism that allows their retrieval by other scientists for such purposes as re­analysis, software and methods development, validation and review’. In 2017, the DDDWG published its final report along with detailed recommendations (https://www.iucr.org/resources/data/dddwg/final-report), a summary of several community-based workshops and publications arising from them. The top two recommendations were as follows:

(i) Authors should provide a permanent and prominent link from their article to the raw data sets which underpin their journal publication and associated database deposition of processed diffraction data (*e.g.* structure factor amplitudes and intensities) and coordinates, and which should obey the ‘FAIR’ principles that their raw diffraction data sets should be Findable, Accessible, Interoperable and Re-usable (https://www.force11.org/group/fairgroup/fairprinciples).

(ii) A registered Digital Object Identifier (doi) should be the persistent identifier of choice (rather than a Uniform Resource Locator, url) as the most sustainable way to identify and locate a raw diffraction data set.

In 2018, the IUCr Commission on Biological Macromolecules (CBM) and the IUCr Committee on Data submitted a memorandum to the IUCr Executive Committee and proposed a mechanism for making diffraction experiments publicly available. The goal of ensuring better reproducibility of scientific discoveries in structural biology would be achieved, in part, by:

(1) Allowing the scientific community to identify and re-use the original diffraction image data from a diffraction experiment, which is the primary source of information used to determine a particular macromolecular structure.

(2) Facilitating structure re-determination using those original diffraction image data.

(3) Providing researchers with a straightforward mechanism that will permit assessing the correctness of the structure determination process.

(4) Providing a mechanism to ensure that the structures in the PDB and the publications derived from them are of the highest possible quality.

IUCr Journals are now taking the lead by encouraging authors to provide a doi for their deposited original raw diffraction data when they submit an article describing a new structure or a new method tested on unpublished diffraction data. In the case of methods developed or tested with raw diffraction data, these data must be available to referees, and deposition of such data will eventually become compulsory. Permanent and prominent links will be provided from articles to the underpinning experimental data of each published research study.

We believe that these actions will maintain crystallography at the forefront of the effort for enhancing transparency and reproducibility of scientific results.

In addition to the references cited above, readers interested in the hows, whys and whats of diffraction data archiving may be referred to the recent in-depth texts by Guss & McMahon (2014 ▸), Kroon-Batenburg & Helliwell (2014 ▸), Kroon-Batenburg *et al.* (2017 ▸), Helliwell *et al.* (2017 ▸), Terwilliger (2014 ▸) and Terwilliger & Bricogne (2014 ▸).
